# Blocking of the Ubiquitin-Proteasome System Prevents Inflammation-Induced Bone Loss by Accelerating M-CSF Receptor c-Fms Degradation in Osteoclast Differentiation

**DOI:** 10.3390/ijms18102054

**Published:** 2017-09-25

**Authors:** Kyunghee Lee, Mi Yeong Kim, Heejin Ahn, Han-Sung Kim, Hong-In Shin, Daewon Jeong

**Affiliations:** 1 Department of Microbiology, Laboratory of Bone Metabolism and Control, Yeungnam University College of Medicine, Daegu 42415, Korea; kyungheelee@ynu.ac.kr (K.L.); gsungmi1004@naver.com (M.Y.K.); fairykd@naver.com (H.A.); 2 Department of Biomedical Engineering, College of Health Science, Institute of Medical Engineering, Yonsei University, Wonju 26493, Korea; hanskim@yonsei.ac.kr; 3 IHBR, Department of Oral Pathology, School of Dentistry, Kyungpook National University, Daegu 41940, Korea; hishin@knu.ac.kr

**Keywords:** proteasome inhibitors, c-Fms, osteoclast, bone resorption

## Abstract

Anti-osteoporotic activity of a blocker of the ubiquitin-proteasome system, bortezomib, has known to be achieved by directly opposed action in increased bone formation by osteoblasts and in decreased bone destruction by osteoclasts. However, the mechanisms underlying the proteasome blocker inhibition of osteoclast differentiation and function are not fully understood. Here, we observed that proteasome inhibitors, such as MG132 and bortezomib, in osteoclasts accelerated the degradation of c-Fms, a cognate receptor of macrophage colony-stimulating factor (M-CSF), and did not affect the amount of receptor activator of nuclear factor kappa-B (RANK), a receptor of receptor activator of nuclear factor kappa-B ligand (RANKL). c-Fms degradation induced by proteasome inhibitors was controlled by the activation of p38/tumor necrosis factor-alpha converting enzyme (TACE)-mediated regulated intramembrane proteolysis (RIPping). This was validated through the restoration of c-Fms using specific inhibitors of p38 and TACE, and a stimulation of p38-dependent TACE. In addition, c-Fms degradation by proteasome inhibition completely blocked M-CSF-mediated intrinsic signalling and led to the suppression of osteoclast differentiation and bone resorption. In a mouse model with intraperitoneal administration of lipopolysaccharide (LPS) that stimulates osteoclast formation and leads to bone loss, proteasome blockers prevented LPS-induced inflammatory bone resorption due to a decrease in the number of c-Fms-positive osteoclasts. Our study showed that accelerating c-Fms proteolysis by proteasome inhibitors may be a therapeutic option for inflammation-induced bone loss.

## 1. Introduction

Macrophage colony-stimulating factor (M-CSF) is a critical cytokine that regulates the survival, proliferation, and the differentiation of monocytes and macrophages [1]. The binding of M-CSF to its receptor c-Fms induces dimerization and autophosphorylation on a tyrosine of the receptor, as well as activates pleiotropic signalling pathways involved in cell spreading, membrane ruffling, and cytoskeleton dynamics [1,2]. c-Fms belongs to the class III receptor tyrosine kinase subfamily, whose members contain a highly glycosylated extracellular domain, single transmembrane domain, and cytoplasmic tyrosine protein kinase domain [3,4]. c-Fms degradation is known to result from lysosomal degradation or a regulated intramembrane proteolysis (RIPping) process [5,6,7,8]. Upon the binding of M-CSF, the c-Fms receptor undergoes internalization and degradation in the lysosome, attenuating or preventing signalling for survival and proliferation [1,9]. Alternatively, c-Fms undergoes a RIPping process through the activation of two consecutive cleavage events, namely ectodomain shedding by tumour necrosis factor-alpha converting enzyme (TACE) and intramembrane cleavage by γ-secretase [6,10]. RIPping of c-Fms can be stimulated by lipopolysaccharide (LPS), 12-*O*-tetradecanoylphorbol-13-acetate (TPA), and bacterial DNA [7].

The M-CSF/c-Fms system has been reported to be involved in various disease processes. Elevated levels of circulating M-CSF were detected in epithelial ovarian and breast cancers, and were associated with a lower survival rate [11,12]. High levels of M-CSF and c-Fms expression were found to correlate with the extent of breast and colorectal cancer metastases, and with histological grades in soft tissue tumours [13,14,15]. Further, M-CSF/c-Fms regulates the development of mammary tumors to malignancy by promoting the infiltration and function of tumour-associated macrophages [16]. The M-CSF/c-Fms system is also known to contribute to the development of inflammatory diseases and atherosclerosis. In a collagen-induced arthritis model, the inhibition of c-M-CSF/c-Fms signalling by two inhibitors, imatinib and GW2580, prevented autoimmune arthritis, macrophage infiltration into synovial joints, osteoclast differentiation, and bone resorption [17]. Additionally, a blockade of the c-Fms signalling pathway by using a monoclonal antibody raised against c-Fms was shown to protect against early-stage atherosclerosis [18].

The ubiquitin-proteasome pathway has been reported to modulate bone metabolism through the induction of osteoblast differentiation and bone formation, as well as the inhibition of osteoclast formation and bone resorption. Inhibition of the proteasome system stimulated osteoblastogenesis and bone formation in calvariae in mice and in multiple myeloma patients, at least in part through the stimulation of bone morphogenetic protein-2 (BMP-2) gene expression and the inhibition of Runx2 proteolytic degradation [19,20,21]. In addition, proteasome inhibitors, namely MG132, MG162, and bortezomib, have been reported to abrogate osteoclastogenesis and bone resorption by inhibiting the receptor of receptor activator of nuclear factor kappa-B ligand (RANKL)-mediated NF-κB activation and NFATc1 expression [22,23,24]. MG132, a peptide aldehyde proteasome inhibitor, has been reported to inhibit, specifically and reversibly, the proteolytic activity of the 26S proteasome complex through covalently binding to the active site of β subunit [25]. As yet, it is unclear whether tuning of the proteasome system can control not only c-Fms degradation during osteoclastogenesis but also bone resorption of c-Fms-positive mature osteoclasts. In this study, we examined the molecular mechanism underlying proteasome inhibitor-induced c-Fms degradation via a RIPping process, and attempted to determine the relationship among the proteasome system, c-Fms regulation, and bone resorption.

## 2. Results

### 2.1. Proteasome Inhibition Suppresses Osteoclast Differentiation by Downregulating M-CSF Receptor c-Fms

**Figure 1:**
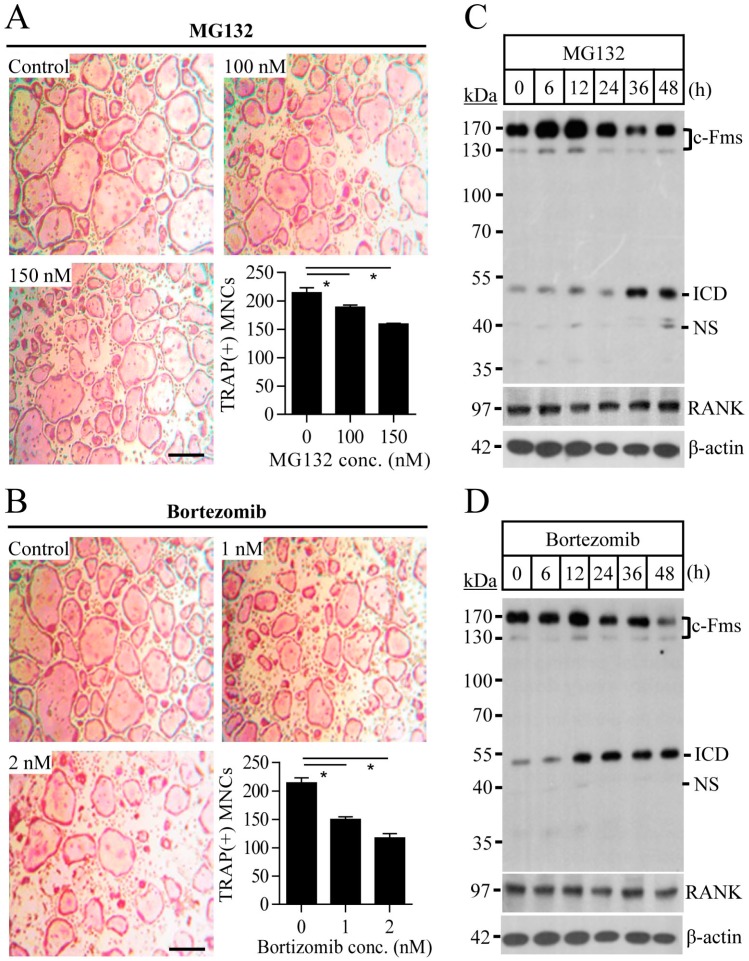
Proteasome inhibitors suppress osteoclast formation and downregulate macrophage colony-stimulating factor (M-CSF) receptor c-Fms. Osteoclast progenitors were cultured with the indicated concentrations of MG132 (**A**) or bortezomib (**B**) in the presence of M-CSF (30 ng/mL) and nuclear factor kappa-B ligand (RANKL) (100 ng/mL) for four days. Tartrate-resistant acid phosphatase (TRAP)-positive multinucleated cells with more than 10 nuclei were counted. Scale bar, 500 µm. For the immunoblot analysis, cells were treated with 150 nM of MG132 (**C**) or 2 nM of bortezomib (**D**). Intracellular domain (ICD) indicates an intracellular domain of c-Fms (~55 kDa) released by intramembrane cleavage, and NS indicates nonspecific bands. Data are representative of three independent experiments. * *p* < 0.01.

**Figure 2:**
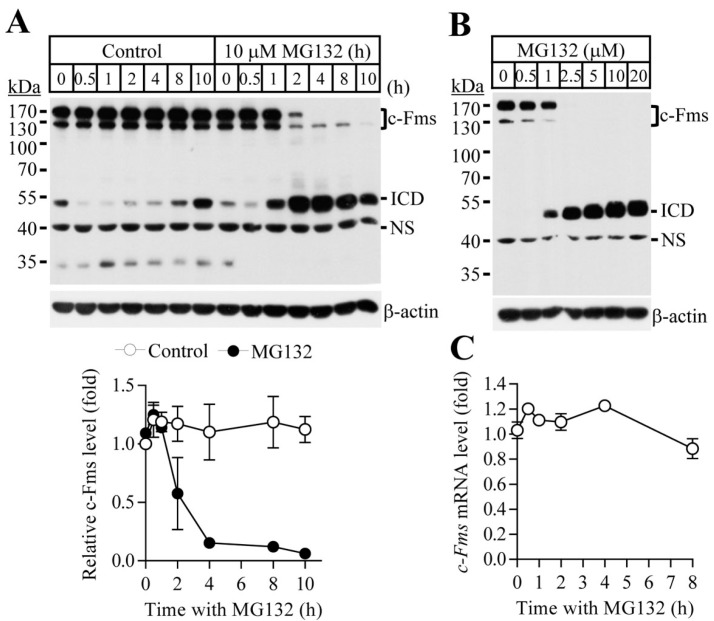
MG132 downregulates the levels of c-Fms protein, but not c-Fms mRNA. Osteoclast progenitors were treated with MG132 (10 µM) for the indicated times (**A**) or with various concentrations of MG132 for 4 h (**B**). ICD, intracellular domain of c-Fms; NS, nonspecific bands; (**C**) cells were treated with MG132 (10 µM) for the indicated times, and relative mRNA levels of c-Fms were analysed by quantitative real-time PCR using GAPDH mRNA as a control.

To study the effect of proteasome inhibition on osteoclast differentiation, osteoclast progenitors were differentiated into osteoclasts by culture with M-CSF and RANKL in the presence or absence of proteasome inhibitors MG132 and bortezomib. Proteasome inhibitors suppressed osteoclast differentiation in a dose-dependent manner, as indicated in the reduced number of tartrate-resistant acid phosphatase (TRAP)-positive multinucleated cells (Figure 1A,B). MG132 (ranging from 0 to 150 nM) and bortezomib (ranging from 0 to 2 nM) did not affect cell proliferation or cause apoptosis of osteoclast progenitors (Figures S1 and S2). M-CSF and RANKL are expressed on osteoblasts and their respective receptors, c-Fms and RANK, are expressed on the surfaces of osteoclasts. RANKL/RANK and M-CSF/c-Fms induce essential osteoclastogenic signalling. To explore the mechanisms underlying the suppressive effect of proteasome inhibitors on osteoclasts differentiation, we first analysed the expression levels of c-Fms and RANK in osteoclast progenitors exposed to proteasome inhibitors. The c-Fms protein level was significantly reduced after MG132 and bortezomib treatment, but that of RANK was not altered (Figure 1C,D). These results suggest that the suppressive effect of proteasome inhibitors on osteoclast differentiation is a result of the downregulation of c-Fms. To definitively determine the mechanism underlying c-Fms downregulation mediated by proteasome inhibitors, it was necessary to define the optimal concentration and time for proteasome inhibitor treatment that resulted in the marked decrease or complete disappearance of c-Fms. In Figure 2A,B showing time- and concentration-dependent c-Fms downregulation by MG132 in osteoclast progenitors, c-Fms protein completely disappeared 4 h after treatment with 10 μM MG132 and was not detected after treatment with 2.5 μM or more concentrations of MG132 for 4 h. Thus, all of the other in vitro experiments were performed with MG132 treatment at a final concentration of 10 μM for 8 h, which showed no significant cytotoxic effects (Figure S3). In contrast with the MG132-induced reduction of c-Fms protein, the c-Fms mRNA expression did not change in response to MG132 (Figure 2C). These data indicate that c-Fms downregulation by proteasome inhibition was a result of post-translational regulation.

### 2.2. Blocking of the Proteasome System Induces c-Fms Degradation by Stimulating p38/TACE-Mediated RIPping

**Figure 3:**
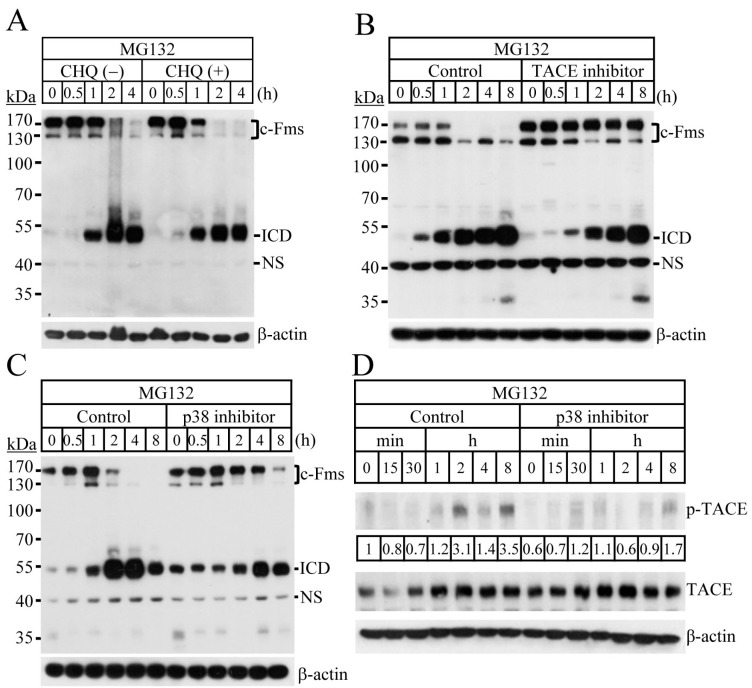
c-Fms is degraded through RIPping induced by p38-mediated tumour necrosis factor-alpha converting enzyme (TACE) activation. Osteoclast progenitors were treated with MG132 (10 µM) in the presence or absence of chloroquine (CHQ, 2 µM, (**A**)), and TAPI-0 (100 µM, (**B**)); (**C**,**D**) osteoclast progenitors were starved of M-CSF, incubated with 20 µM SB203580 (a specific inhibitor of p38) for 30 min, and then treated with MG132 (10 µM). Fold changes of phosphorylated-TACE (p-TACE) were presented. ICD, intracellular domain of c-Fms; NS, nonspecific bands.

Degradation of c-Fms has been reported to occur through two main pathways: intralysosomal degradation of the receptor-ligand complex, and the TACE-dependent RIPping process [5,7]. To determine the degradation pathway of c-Fms induced by proteasome inhibitors, we analysed the effect of the lysosomal inhibitor chloroquine on MG132-induced c-Fms degradation. Chloroquine treatment did not alter the pattern of c-Fms degradation by MG132 (Figure 3A). The RIPping process of c-Fms has recently been reported to include two consecutive proteolytic cleavages, ectodomain shedding by TACE, and intramembrane cleavage by γ-secretase [10]. Intramembrane cleavage leads to the release of the intracellular domain (ICD), which corresponds to a 55-kDa protein in the cytosol [6]. In Figure 2A,B, c-Fms protein (immature and mature forms) decreased and ICD fragments increased simultaneously after treatment with proteasome inhibitors. Inactivation of TACE, the first proteolytic enzyme of the RIPping process by TAPI-0 completely blocked c-Fms degradation by MG132 (Figure 3B). These results clearly indicate that c-Fms degradation by MG132 is mediated by RIPping, and not through the lysosomal degradation pathway. RIPping of c-Fms has been reported to be associated with the MAPKs and PKC signalling pathways [7,10]. To assess the signalling pathways involved in c-Fms degradation by proteasome inhibitors, we next analysed the activities of MAPKs in response to MG132. MG132 treatment resulted in the activation of all three MAPKs: ERK, JNK, and p38 (Figure S4).

Using specific inhibitors, we showed that MG132-induced c-Fms degradation via the RIPping process was suppressed by p38 inactivation, but not by the inactivation of ERK, JNK, PKCα, and PKCδ (Figure 3C and Figure S5). To analyse the relationship between p38 and TACE activation in the MG132-induced c-Fms RIPping process, osteoclast progenitors were treated with MG132 in the presence or absence of a specific p38 inhibitor, and the activity of TACE was measured. Inactivation of p38 suppressed MG132-induced TACE activation (Figure 3D). Together, these results indicate that c-Fms degradation by MG132 is mainly achieved through RIPping by activating p38-mediated TACE signalling.

### 2.3. Proteasome Inhibition Suppresses M-CSF/c-Fms-Mediated Intrinsic Signalling and Bone Resorption Activity of Mature Osteoclasts

**Figure 4:**
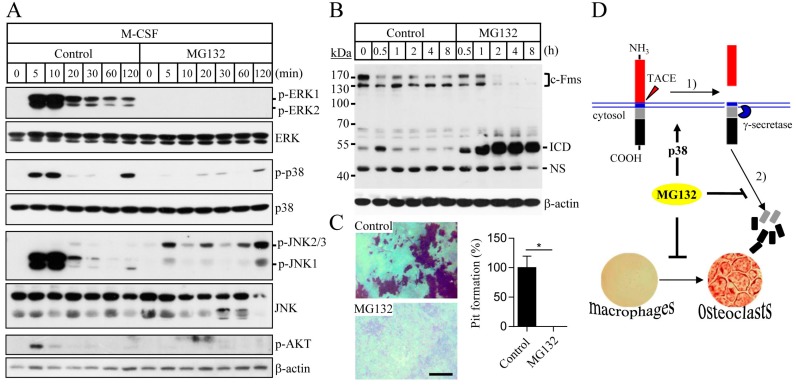
A proteasome inhibitor blocks M-CSF-induced intrinsic signaling and osteoclastic bone resorption. (**A**) Osteoclast progenitors were incubated with MG132 (10 µM) for 4 h in the absence of M-CSF and then were treated with M-CSF (30 ng/mL); (**B**) osteoclast progenitors were cultured with M-CSF and RANKL for 4 days. The resulting mature osteoclasts were starved of M-CSF and RANKL, and then treated with MG132 (10 µM). ICD, intracellular domain of c-Fms; NS, nonspecific bands; (**C**) mature osteoclasts were grown on dentin discs with M-CSF and RANKL, in the presence or absence of MG132 (10 µM) for 24 h. The areas of resorbed pits were measured using Image-Pro Plus version 6.0. * *p* < 0.01. Scale bar, 100 µm; (**D**) proposed schematic representation of the TACE-mediated c-Fms RIPping process induced by proteasome inhibitors. Sequential activation of p38 and TACE by proteasome inhibitors accelerates RIPping of c-Fms and leads to an inhibition of osteoclast differentiation.

The binding of M-CSF to its cognate receptor c-Fms is known to mediate the activation of MAPKs and Akt signalling, which are essential for the osteoclast differentiation and function [26]. M-CSF, together with RANKL, plays an important role in the survival of mature osteoclasts and bone resorption. To examine the effect of MG132 on M-CSF/c-Fms signalling, osteoclast progenitors were pretreated with MG132, followed by the stimulation with M-CSF. MG132 treatment suppressed M-CSF-induced activation of MAPKs and Akt (Figure 4A). These findings indicate that MG132 treatment can inhibit osteoclast differentiation by blocking M-CSF/c-Fms-mediated intrinsic signalling. To further explore the effect of proteasome inhibition on the activity of osteoclasts, we analysed c-Fms degradation in mature osteoclasts that can resorb the bone. The pattern of c-Fms degradation in mature osteoclasts was similar to that of osteoclast progenitors (Figure 4B). We next evaluated the effect of MG132 on bone resorption by mature osteoclasts. After seeding the mature osteoclasts on dentin discs, cells were treated with MG132 in the presence of M-CSF and RANKL, and the formation of resorption pits was analysed. MG132 treatment caused a marked reduction in osteoclastic resorptive activity (Figure 4C), indicating that tuning of the proteasome system can control osteoclast function.

### 2.4. Proteasome Inhibition Prevents Inflammation-Induced Osteoporosis in Mice

**Figure 5:**
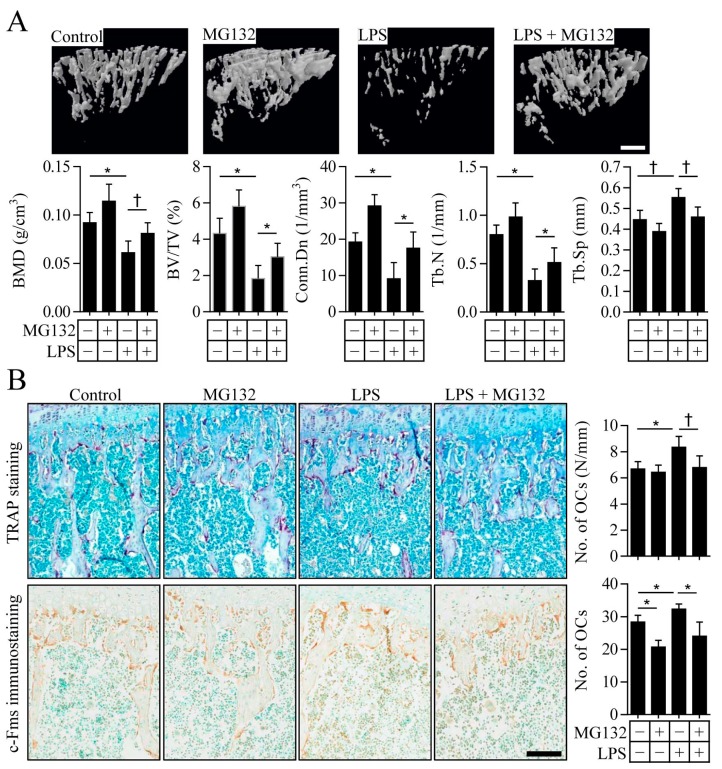
MG132 inhibits LPS-induced osteoporotic bone loss in mice. (**A**) Representative µCT images of tibial trabecular bone, as well as bone loss indices, were analysed in mice injected intraperitoneally with MG132 and/or LPS. Quantitative data for trabecular structural parameters are also shown as means ± SD (*n* = 7 mice per group). Scale bar, 10 µm; (**B**) tibial sections from mice treated as in (**A**) were stained with TRAP (upper images) to detect osteoclasts and subjected to immunohistochemistry to visualize c-Fms-positive osteoclasts (lower images). The number of osteoclasts per millimeter of trabecular bone surface and the number of c-Fms-positive osteoclasts in a defined trabecular bone area were counted. All quantitative data are presented as means ± SD (*n* = 7) * *p* < 0.01, † *p* < 0.05. Scale bar, 200 µm.

Based on the results of our in vitro experiments, we next explored the effect of proteasome inhibitors on inflammation-induced osteoporosis. Intraperitoneal injection of LPS as an inducer of inflammation in mice resulted in an osteoporotic phenotype characterized by a decreased bone mineral density (BMD), bone volume (BV/TV), connectivity density, and trabecular number, as well as an increase in trabecular separation (Figure 5A). The administration of MG132 ameliorated LPS-induced osteoporosis in mice, indicating that MG132 could prevent LPS-induced bone loss (Figure 5A). Interestingly, treatment with MG132 alone caused a slight increase in BMD, BV/TV, connectivity density, and trabecular number, although these increases were not statistically significant, suggesting that MG132 itself might have a stimulatory effect on bone formation. To identify the changes in the number of osteoclasts on trabecular bone surfaces, bone histomorphometry was performed. Analysis of osteoclasts by TRAP staining revealed that the LPS-induced increase in the number of TRAP-positive osteoclasts was prevented by treatment with MG132 (Figure 5B, upper panel). Further immunohistochemical studies showed significantly lower levels of c-Fms-positive osteoclasts on the trabecular bone surface of mice treated with MG132 when compared with those of control-treated mice (Figure 5B, lower panel). c-Fms immunoreactivity was increased in LPS-treated mice, but was significantly decreased in mice treated with both MG132 and LPS. These results indicate that MG132 treatment can directly regulate osteoclast differentiation and prevent bone loss induced by LPS, at least in part through the suppression of c-Fms expression.

## 3. Discussion

In the present study, we showed that proteasome inhibitors could suppress osteoclast differentiation by stimulating p38/TACE-mediated RIPping of c-Fms (Figure 4D). The ubiquitin-proteasome pathway that selectively degrades unfolded or damaged proteins has been reported to play an important role in the development and progression of cancer, as well as the regulation of bone metabolism [27,28]. Multiple myeloma is a type of cancer formed from bone marrow plasma cells that show a high capacity for osteolytic bone destruction [29]. Most patients with multiple myeloma have skeletal complications [27,30]. Specifically, multiple myelomacells have been reported to produce both osteoclastic stimulatory factors such as RANKL, TNF-α, MIP-1α, and IL-6, and osteoblastic inhibitory factors, including DKK1, sclerostin, hepatocyte growth factor, and TGF-β [31]. The proteasome inhibitor bortezomib has shown efficacy in suppressing multiple myeloma bone disease, as well as treating multiple myeloma [32,33]. Further, proteasome inhibitors have shown a potent suppressive effect on multiple myeloma bone disease, increasing the osteoblast activity and inhibiting the osteoclast activity both in vitro and in vivo [21,24,34]. Proteasome inhibitors were found to stimulate osteoblast differentiation by inducing BMP-2 expression and blocking proteolytic degradation of Runx2 [19,20]. On the other hand, bortezomib and MG132 have been reported to inhibit osteoclast differentiation by downregulating RANKL-dependent activity such as NF-κB activation, NFATc1 signalling, and TRAF6 production [22,23,35]. Here, we report that the suppressive action of proteasome inhibitors during osteoclastogenesis is associated with the regulation of M-CSF receptor c-Fms, which is expressed on osteoclasts’ plasma membranes.

In this study, we showed that proteasome inhibitor-mediated regulation of TACE activity for ectodomain shedding of c-Fms was associated with p38 signalling, but not with ERK or PKC signalling. TACE, also known as ADAM17, has been identified as a metalloprotease that acts in ectodomain shedding [36]. TACE has also been reported to mediate cleavage of the membrane-proximal extracellular domain of a number of its substrates, including immunoregulatory cytokines, cell adhesion molecules, growth factors, and receptors [37,38], with its activation dependent on ERK, p38, and PKC signalling [39,40,41]. In breast carcinoma cells, ERK or p38 signalling induces TACE activation by increasing the presentation of TACE on cell surfaces and decreasing the inhibition of TACE activation by tissue inhibitor of metalloproteinase-3 (TIMP3) [39]. Activated p38 was shown to directly phosphorylate TACE on Thr735, which is a critical site associated with TACE-mediated ectodomain shedding of TGF-α ligands [40]. TACE activity for ectodomain shedding of L-selectin was found to be regulated by p38 and PKC signalling in primary murine leucocytes [41]. Considering that TACE is expressed ubiquitously in animal or human tissues and is an enzyme with many substrates [36,38], the regulatory mechanisms of TACE activation could be different in various cell types and environmental conditions.

In addition to critical roles in osteoclast differentiation and survival [1], the M-CSF/c-Fms system is an essential regulator of survival, proliferation, differentiation, and function of mononuclear phagocytes and macrophages [42]. Mononuclear phagocytes and their terminally differentiated cells, macrophages, have been reported to play an important role in innate and acquired immunity, tissue homeostasis, and inflammation [43]. Inflammatory cytokines that are highly produced by macrophages play critical roles in sepsis, rheumatoid arthritis, inflammatory bowel disease, and bone defects [44,45,46]. Because macrophages are central effector cells of the innate immune system and are capable of differentiating into osteoclasts [47], the function of macrophages must be tightly regulated in bone remodelling. Myeloma cells, malignant cells arising from normal plasma cells, produce various inflammatory cytokines, including osteoclastic stimulatory factors and osteoblastic inhibitory factors, suggesting that multiple myeloma bone disease could be considered as an inflammatory bone disease [31]. Our study provided the opportunity to demonstrate the functional role of proteasome inhibitors in an inflammation-induced bone defect model and revealed that the proteasome inhibitor MG132 prevents LPS-induced osteoporotic bone loss, which results from a decreased number of c-Fms-positive osteoclasts on the surface of the trabecular bone. Thus, a reduction in c-Fms-positive osteoclasts by proteasome inhibitors might be an important target for the treatment of inflammation-induced bone loss in both an LPS-driven sepsis model and multiple myeloma. Interestingly, MG132 has been reported to promote osteocytogenesis [48] and osteocytes have been shown to be the critical source of RANKL [49], suggesting the stimulatory effect of MG132 on osteoclastogenesis. In our study, the preventive action of MG132 on bone loss in LPS-treated mouse was thought to be mainly caused by the inhibitory effect of MG132 on osteoclastogenesis rather than by its stimulatory effect.

In conclusion, we showed that proteasome inactivation inhibits the M-CSF-mediated intrinsic signalling from macrophages via the accelerated proteolytic degradation of M-CSF receptor c-Fms and subsequently blocks the differentiation of osteoclast progenitor macrophages into osteoclasts and osteoclastic bone resorption. This study on the regulatory mechanisms underlying c-Fms RIPping induced by proteasome inhibitors extends our knowledge of macrophage behaviour and bone physiology, which may facilitate the identification of new therapeutic targets for the treatment of not only inflammation-induced bone loss but also macrophage-mediated inflammatory diseases.

## 4. Materials and Methods

### 4.1. Osteoclast Progenitor Preparation and Osteoclast Differentiation

Bone marrow-derived mononuclear cells were isolated from the tibias and femurs of 6-week-old male C57BL/6J mice (Central lab animal, Seoul, Korea) by flushing the bone marrow cavity. Erythrocytes were lysed in red blood cell lysing buffer (Sigma, St. Louis, MO, USA), and the remaining cells were cultured in α-minimum essential medium (α-MEM; Invitrogen, Carlsbad, CA, USA) supplemented with antibiotics, 10% fetal bovine serum, and recombinant human M-CSF (5 ng/mL) overnight. Non-adherent cells that included monocytes were harvested and cultured in α-MEM with M-CSF (30 ng/mL) for 3 days to induce osteoclast progenitors. For osteoclast differentiation, osteoclast progenitors (1 × 10^4^ cells per well in 48-well culture plates) were further cultured in the presence of M-CSF (30 ng/mL) and RANKL (100 ng/mL) for four days. To identify osteoclasts, TRAP staining was performed using an Acid Phosphatase Kit (Sigma) according to the manufacturer’s instructions. TRAP-positive cells with 10 or more nuclei were counted under a light microscope. All of the animal studies were reviewed and approved by the institutional review board of Yeungnam University Medical Center (24 February 2016; YUMC-AEC2015-041) and were performed in accordance with the Guide for the Care and Use of Laboratory Animals.

### 4.2. Pit Formation Assay

For the bone resorption assays, mature osteoclasts were seeded on dentin discs (IDS Ltd., Boldon, UK) and incubated with M-CSF and RANKL in the presence or absence of MG132 for 24 h. Cells attached to dentin slices were removed by sonication, and the resorption pits were visualized using 1% toluidine blue (Sigma). Photographs of the resorption pits were taken under a light microscope, and the resorption areas were measured using Image-Pro Plus version 6.0 (Media Cybernetics, Rockville, MD, USA).

### 4.3. Immunoblot Analysis

For the immunoblot analysis, cells were starved of M-CSF for 4 h and treated with MG132 or bortezomib (Sigma) and then dissolved in DMSO. Cells were then lysed using a buffer containing 20 mM Tris-HCl, pH 7.5, 150 mM NaCl, 1% Nonidet P-40, 0.5% sodium deoxycholate, 1 mM ethylenediaminetetraacetic acid 0.1% sodium dodecyl sulfate (SDS), and protease inhibitors (Complete Protease Cocktail Inhibitor Tablets, Roche Molecular Biochemicals, Munich, Germany). Protein concentrations were determined using a DC protein assay (Bio-Rad, Hercules, CA, USA). Whole cell extracts were fractionated by SDS-polyacrylamide gel electrophoresis and then transferred to a nitrocellulose membrane (GE Healthcare, Pittsburgh, PA, USA). Membranes were incubated with primary antibodies (1:1000) against c-Fms, RANK, p-TACE, TACE, p-ERK, ERK, p-p38, p38, p-JNK, JNK, p-Akt (Cell Signaling Technology, Frankfurt, Germany), or β-actin as a loading control (Santa Cruz Biotechnology, Santa Cruz, CA, USA). Horseradish peroxidase-conjugated secondary antibodies and enhanced chemiluminescence (ECL) reagents (Abfrontier, Seoul, Korea) were used for the detection of immune complexes.

### 4.4. Quantitative Real-Time PCR

For real-time quantitative polymerase chain reaction (PCR) analysis, total RNA was prepared using Trizol reagent (Invitrogen), and 2 µg of RNA were reverse transcribed using an M-MLV Reverse Transcription Kit (Invitrogen). Real-time PCR analysis was performed using a SYBR Premix Ex Taq (Takara Bio, Shiga, Japan) and Applied Biosystems 7500 Sequence Detection System (Foster City, CA, USA). To determine the relative gene expression levels, the comparative threshold cycle method was used with glyceraldehyde-3-phosphate dehydrogenase (GAPDH) mRNA as a housekeeping standard. PCR primers were synthesized by Bionics (Seoul, Korea), and their sequences were as follows: mouse c-Fms: forward 5′-CAG AGC CCC CAC AGA TAA AA-3′ and reverse 5′-GTC CAC AGC GTT GAG ACT-3′; mouse GAPDH: forward 5′- AGG TCG GTG TGA ACG GAT TTG-3′ and reverse 5′-TGT AGA CCA TGT AGT TGA GGT-3′.

### 4.5. Cell Proliferation and Apoptosis Assay

For all of the cell proliferation assays, osteoclast progenitors (1 × 10^5^ cells per well in 12-well culture plates) were incubated with M-CSF (30 ng/mL) in the presence or absence of proteasome inhibitors for the indicated times. Cell proliferation was measured by cell counting with a haemocytometer after staining with Trypan blue. For the apoptosis assay, cells were cultured and then stained using an Apo-BrdU In Situ DNA Fragmentation Assay Kit (BioVision, Milpitas, CA, USA) according to the manufacturer's instructions. The fluorescence-stained cells were examined for apoptosis under a fluorescence microscope.

### 4.6. Mouse Model with Inflammation-Induced Bone Loss

For an analysis of the effect of MG132 on animal models, 7-week-old C57BL/6J male mice were injected intraperitoneally with LPS (5 mg/kg, Sigma), MG132 (2.5 mg/kg), or PBS (control) on days 0, 2, and 4. Mice were then sacrificed on day six to analyse trabecular morphometry and bone histology. To assess trabecular morphometry, the proximal tibia was scanned in three dimensions by high-resolution micro-computed tomography (1076 μCT System; Skyscan, Aartselaar, Belgium). The μCT analysis was performed at an isotropic resolution of 18 μm and the analysis of trabecular bone in the metaphyseal region was confined to 1.0 mm adjacent to the growth plate. Bone indices such as bone mineral density (BMD), bone volume as a percentage of total bone volume (BV/TV), connectivity density (Conn.Dn), trabecular number (Tb.N), and trabecular separation (Tb.Sp) were measured by microcomputed tomography (μCT). To analyse bone histomorphometry, a 5-μm-thick sagittal section was stained with TRAP to detect osteoclasts and was counterstained with methylene blue. The number of osteoclasts was measured in the area of 0.59 mm^2^. Immunohistochemical staining was performed using the ImmunoCruz rabbit ABC Staining System (Santa Cruz Biotechnology) for c-Fms with polyclonal rabbit-anti-c-Fms antibody (1:200) according to the manufacturer’s instructions. The numbers of osteoclasts and c-Fms-positive osteoclasts on the surface of trabecular bone were counted under a light microscope.

### 4.7. Statistical Analysis

Quantitative data are expressed as the mean ± SD from at least three independent experiments. The means of two groups were compared using the Student’s two-tailed *t*-test with a 95% confidence interval. For statistical analyses with multiple comparisons between two groups, we used ananalysis of variance (ANOVA) and *post-hoc* tests in the SPSS 21.0 software package. A *p* value less than 0.05 was considered statistically significant.

## Supplementary Materials

Supplementary materials can be found at www.mdpi.com/1422-0067/18/10/2054/s1.
