# Supplementary material for: Blocking of the Ubiquitin-Proteasome System Prevents Inflammation-Induced Bone Loss by Accelerating M-CSF Receptor c-Fms Degradation in Osteoclast Differentiation
Source: Int J Mol Sci. 2017 Sep 25;18(10):2054. doi: 10.3390/ijms18102054 (PMC5666736; doi:10.3390/ijms18102054)
Supplement: Supplementary file 1 [file ijms-18-02054-s001.pdf]

Supplementary Figures

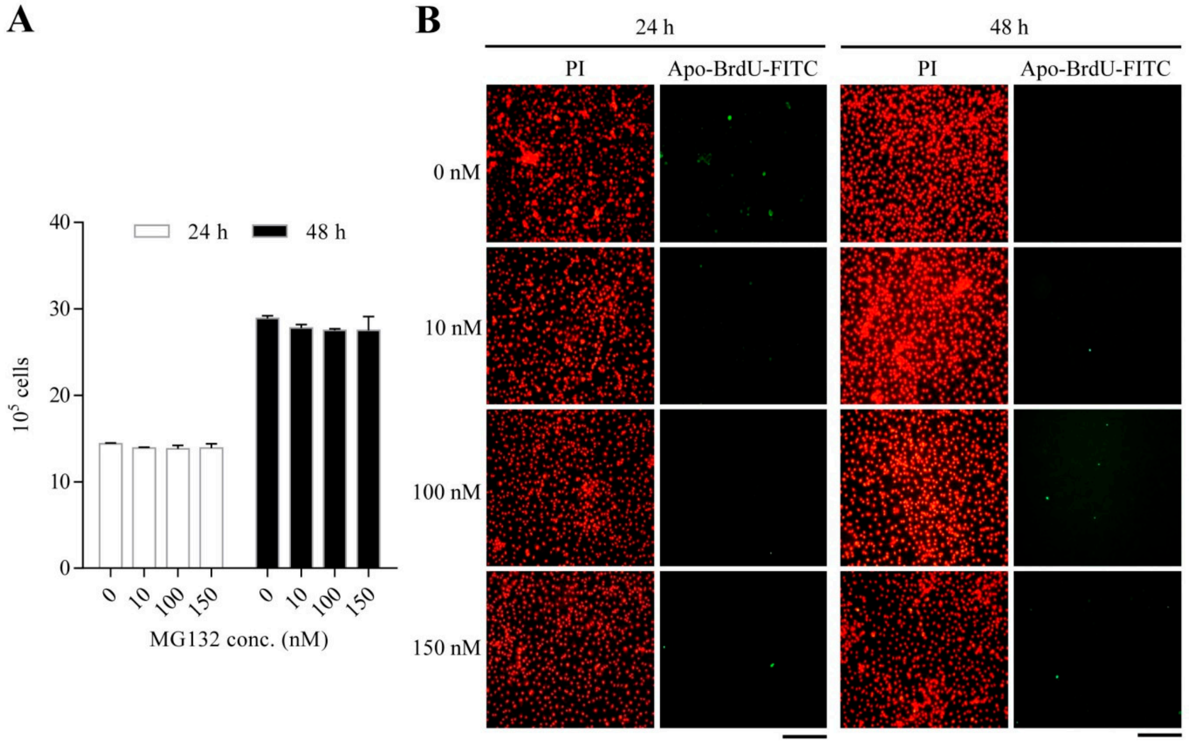

Figure S1. Effects of MG132 on cell proliferation and apoptotic cell death. Osteoclast progenitors were treated with the indicated concentrations of MG132 for 24 h or 48 h in the presence of M-CSF (30 ng/ml). (**A**) Cell proliferation was determined by counting the number of cells with a haemocytometer after trypan blue staining. Quantitative data are presented as means ± SD (n = 3) of three independent experiments. (**B**) Apoptotic cell death (shown in green) was identified by DNA breaks using an Apo-BrdU In Situ DNA Fragmentation Assay Kit. Red indicates propidium iodide (PI)-stained cells. Images are representative of three independent experiments. Scale bar, 100 μm.

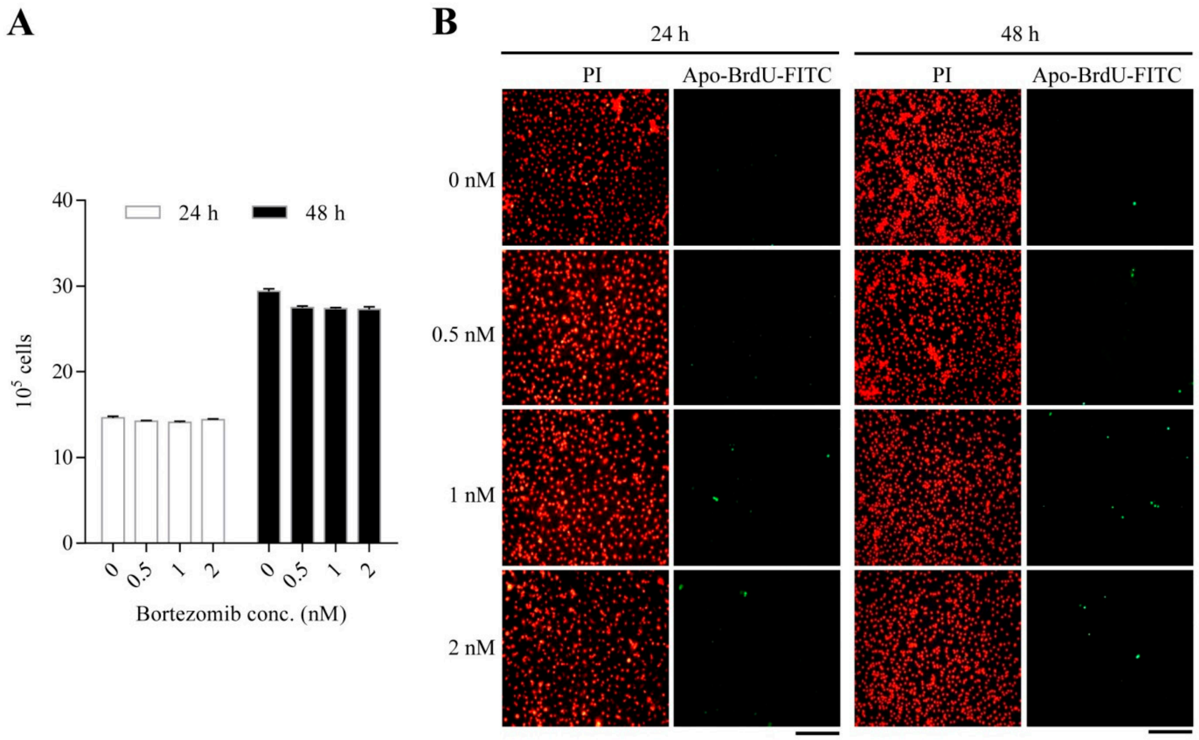

Figure S2. Effects of bortezomib on cell proliferation and apoptosis. After osteoclast progenitors were treated with the indicated concentrations of bortezomib for 24 h or 48 h in the presence of M-CSF (30 ng/ml), cell proliferation (**A**) and apoptosis (**B**, green color) were identified as described in Fig. S1. Red indicates propidium iodide (PI)-stained cells. Quantitative data are presented as means ± SD (n = 3) of three independent experiments. Images are representative of three independent experiments. Scale bar, 100 μm.

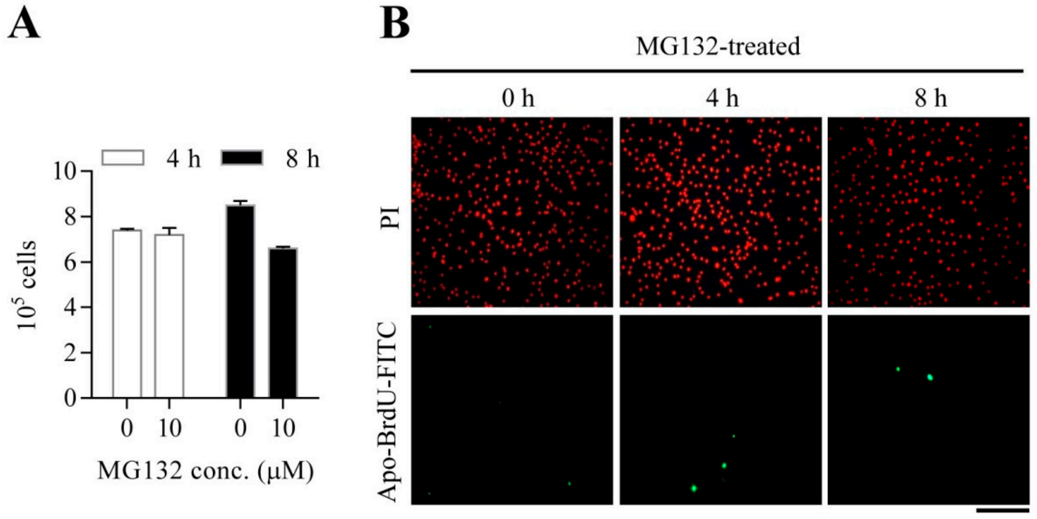

Figure S3. Effects of MG132 (10 μM) on cell proliferation and apoptosis. After osteoclast progenitors were treated with MG132 (10 μM) for 4 h or 8 h in the presence of M-CSF (30 ng/ml), cell proliferation (**A**) and apoptosis (**B**, green) were assessed as in Fig. S1. Red represents propidium iodide (PI)-stained cells. Quantitative data are presented as means ± SD (n = 3) of three independent experiments. Images are representative of three independent experiments. Scale bar, 100 μm.

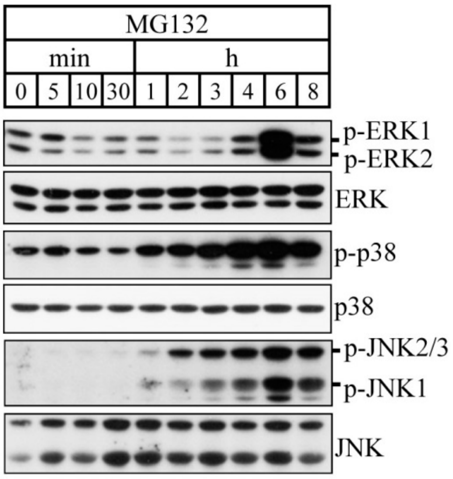

Figure S4. MAPKs (ERK, JNK, and p38) activation by MG132. Osteoclast progenitors starved of M-CSF for 4 h were cultured with MG132 (10 μM) for the indicated times, and whole cell lysates were subjected to immunoblot analysis.

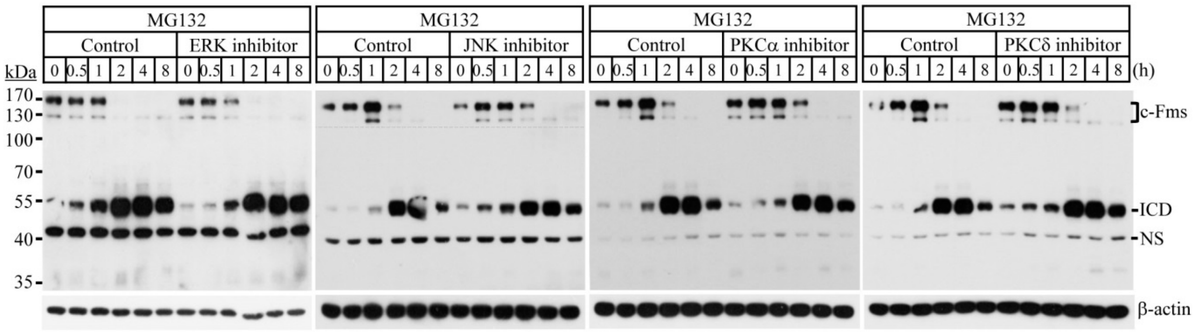

Figure S5. MG132-induced c-Fms degradation is independent of ERK, JNK, PKCα, and PKCδ signalling. Osteoclast progenitors were incubated with PD98059 (10 µM; a specific inhibitor of ERK), SP600125 (20 µM; a specific inhibitor of JNK), Gö6976 (1 µM; a specific inhibitor of PKCα), and rottlerin (1 µM; a specific inhibitor of PKCδ) for 30 min. Cells were then treated with MG132 (10 µM) for the indicated times. Total cell lysates were subjected to immunoblotting with antibodies against c-Fms or β-actin (loading control). ICD, intracellular domain of c-Fms; NS, nonspecific bands. Data are representative of three independent experiments.
